# Contrasted agronomical and physiological responses of five *Coffea arabica* genotypes under soil water deficit in field conditions

**DOI:** 10.3389/fpls.2024.1443900

**Published:** 2024-10-08

**Authors:** Thuan Sarzynski, Philippe Vaast, Clément Rigal, Pierre Marraccini, Boris Delahaie, Frédéric Georget, Chang Thi Quynh Nguyen, Hung Phi Nguyen, Hai Thi Thanh Nguyen, Quyen Luu Ngoc, Giang Khong Ngan, Laurent Bossolasco, Hervé Etienne

**Affiliations:** ^1^ CIRAD (Centre de Coopération Internationale en Recherche Agronomique Pour le Développement), UMR DIADE, Montpellier, France; ^2^ UMR DIADE (Diversity, Adaptation, Development of Plants), University of Montpellier, CIRAD, IRD, Montpellier, France; ^3^ ECOM-SMS (Sustainable Management Services ECOM Agroindustrial), Ho Chi Minh City, Vietnam; ^4^ UMR Eco & Sols, CIRAD, Montpellier, France; ^5^ ICRAF, Vietnam Office, Hanoi, Vietnam; ^6^ CIRAD UMR ABSYS, Montpellier, France; ^7^ ABSYS, Université Montpellier, CIRAD, INRAE, Supagro, Montpellier, France; ^8^ NOMAFSI (Northern Mountainous Agriculture Forestry Science Institute) Mai Son Research Centre, Son La, Vietnam; ^9^ NOMAFSI Headquarter, Hanoi, Vietnam; ^10^ AGI (Agriculture Genetics Institute), Hanoi, Vietnam

**Keywords:** climate change, Coffea arabica, *drought tolerance*, evapotranspiration, photosynthesis, plant physiology, Vietnam, water relations

## Abstract

**Introduction:**

Breeding programs have developed high-yielding Coffea arabica F1-hybrids as an adaptation against adverse conditions associated with climate change. However, theresponse to drought of coffee F1 hybrids has seldom been assessed.

**Methods:**

A trial was established with five C. arabica genotypes (2 pure lines: Catimor and Marsellesa and 3 F1 hybrids: Starmaya, Centroamericano and Mundo Maya) planted under the leguminous tree species Leuceana leucocephala. Coffee growth, yield and physiological responses were assessed under a rain-fed (control: CON) and a rainfall reduction treatment (RR) for 2 years.

**Results:**

The RR treatment created a long-term rainfall deficit in a region with suboptimal temperature similar to those predicted by climate change scenarios. Moreover, the RR treatment reduced soil water content by 14% over 2 successive years of production and increased hydric stress of the three F1-hybrids (leaf water potentials averaged -0.8 MPa under RR compared with -0.4 MPa under CON). Under RR, coffee yields were reduced from 16 to 75% compared to CON. Mundo Maya F1 hybrid was the sole high-yielding genotype apable of sustaining its yield under RR conditions. Our results suggested that its significant increase in fine root density (CON = 300 and RR = 910 root.m-2) and its maintenance of photosynthetic rate (2.5 – 3.5 mmol CO2 m-2 s-1) at high evaporative demand might explain why this genotype maintained high yield under RR condition.

**Discussion:**

This work highlights a possible drought tolerance mechanism in fruit bearing adult coffee trees where the plant fine root number increases to intake more water in order to preserve turgor and sustainphotosynthesis at high ETo and therefore conserves high yield in dry conditions.

## 1 Introduction

Coffee provides a livelihood for over 12 million households in tropical countries such as Brazil, Vietnam, Colombia and Indonesia (Pham et al., 2019; Pappo et al., 2021). As for many other crops, the two main *Coffea* species, *C. arabica* (60% of traded coffee) and *C. canephora* (40%), are both sensitive to environmental changes caused by climate change (DaMatta and Ramalho, 2006; Dinh et al., 2022; Kath et al., 2022). Climate change leads to increased average temperatures and changes in rain patterns across the seasons worldwide. Precipitation is the main environmental factors influencing soil water availability to plants and thereby their productivity while temperature has a secondary importance in changing soil water availability but still highly influence plant physiology and productivity (Ray et al., 2015; Hatfield et al., 2020). In this context, global models predicted a decrease in the range of 20-55% of the suitable coffee cultivation area due to climate change (Bunn et al., 2015; Ovalle-Rivera et al., 2015; Magrach and Ghazoul, 2015; Grüter et al., 2022).

Seasonal drought naturally occurs in most tropical agroecosystems where coffee is cultivated, and comes along with lower soil water content and high evaporative demand. These conditions lead to coffee plants entering a state of dormancy combining low physiological activity and low vegetative and reproductive growth (Novaes et al., 2010; Almeida et al., 2021). The first rain or first round of irrigation after a dry period breaks coffee plant dormancy and triggers flowering (Carr, 2001). Yet, climate change will prolong the period of drought and make water deficit and evaporative demand more extreme, thereby affecting coffee development, flowering potential and yield (Kath et al., 2022).

Reviews have already been published on the effect of drought on coffee physiology and agronomy (Carr, 2001, 2012; DaMatta and Ramalho, 2006; DaMatta et al., 2018). A lower soil water content has been reported to decrease coffee vegetative growth (Cai et al., 2007; Chemura, 2014; Erdiansyah et al., 2019; Kiwuka et al., 2022), production (Rakocevic et al., 2023), bean size and biochemical compounds (Vinecky et al., 2017) and sensory quality (Tesfaye et al., 2013; Ferreira et al., 2021a). Lower soil water content also decreases coffee leaf water potential (Gutierrez and Meinzer, 1994; Pinheiro et al., 2005; Padovan et al., 2018; Martins et al., 2019), stomatal conductance (Pinheiro et al., 2005; Cai et al., 2007; Tesfaye et al., 2013; Martins et al., 2019), photosynthetic rate (Cai et al., 2007; Franck and Vaast, 2009; Martins et al., 2019; Almeida et al., 2021) and coffee tree transpiration (DaMatta and Ramalho, 2006; van Kanten and Vaast, 2006; Venturin et al., 2020). Facing these effects, the development of new coffee varieties better adapted to climate change is now one of the main goals of coffee breeding programs (Bertrand et al., 2019).

Past coffee plant breeding programs have produced coffee genotypes with higher yields and higher resistance to pests and diseases (mainly leaf rust), but they rarely intended to produce genotypes adapted to the drier and hotter environments caused by climate change (van der Vossen et al., 2015; Bertrand et al., 2019). It is only in the last decade that breeders have realized the imminent challenges posed by climate change and identified key traits for breeding climate change-adapted Arabica genotypes. Drought tolerance and the associated agronomical and physiological traits are systematically sought after in new breeding programs (Breitler et al., 2022; Silva et al., 2022). Arabica coffee F1-hybrids generated in the 2000s by crossing wild Ethiopian Arabica with commercial American lines have shown a higher vegetative development and yield as well as a higher homeostasis to environmental stress than pure lines across a range of monoculture and agroforestry systems in Central America (Bertrand et al., 2005; Bertrand and Lara-Estrada, 2011; Georget et al., 2019; Marie et al., 2020; Pappo et al., 2021). Recently, studies have highlighted a higher adaptation of Arabica F1-hybrids to low light environment linked to a high photosynthetic electron transport chain efficiency and chlorophyll a fluorescence (Toniutti et al., 2019) as well as a high content of 5-caffeoylquinic acid (CQA) and mangiferin (Duangsodsri et al., 2020). However, the specific physiological aspects involved in their superior performance and homeostasis, notably their response to drought, remain to be fully understood.

Drought tolerance has been defined as the maintenance of an agronomic trait (Erdiansyah et al., 2019; Silva et al., 2022) or a physiological trait (DaMatta, 2004; Pinheiro et al., 2005; Martins et al., 2019) in conditions of soil water deficit and high evaporative demand (Gambetta et al., 2020). Field and controlled experiments have highlighted the importance of genotypic diversity in influencing the diverse physiological and agronomical responses to soil water deficit. The location of origin of wild *C. canephora* trees was reported to predict well their potential for drought tolerance (Kiwuka et al., 2022; de Aquino et al., 2022). In *C. canephora*, clones able to maintain high yield under drought (drought tolerant) showed a lower stomatal conductance (*g_s_*) and a longer and denser root system than drought sensitive clones when exposed to soil water deficit (Pinheiro et al., 2005). In the main reference study on *C. arabica*, drought tolerance was defined by a high predawn leaf water potential in dry conditions and was associated with tall genotypes with high sapwood and leaf area, and low *g_s_* (Tausend et al., 2000).

Experiments testing coffee drought tolerance are dominated by two experimental systems, namely water suppression in pots in greenhouse conditions or irrigation experiments in field conditions. Water suppression in pots simulates an extreme and quick drought stress where young coffee plants are subject to xylem near-embolism and thereby decrease their water potential, and lose turgor and leaves within a few days (Kiwuka et al., 2022). In field experiments, irrigated blocks are compared with control blocks without irrigation. In fact, such experiments illustrate the effect of irrigation and soil water availability rather than the effect of drought (Tesfaye et al., 2013; Vinecky et al., 2017; Ferreira et al., 2021b; Rakocevic et al., 2023). Moreover, most drought experiments, whether in pots or in irrigated trials, have looked at the effect of drought on juvenile non-producing coffee plants (Novaes et al., 2010; Chemura, 2014; Erdiansyah et al., 2019) and did not assess coffee production, the ultimate key variable when defining coffee drought tolerance. Such experiments on immature plants disregarded the well-known effect of sink-source interaction in regulating coffee physiology and water relations as the tree is bearing fruits and carbohydrates demand in these sink organs increases (Vaast et al., 2006; Morais et al., 2012; Almeida et al., 2021). A third experimental design, rarely used in coffee, partially solves these shortcomings (Pappo et al., 2021). It consists in enhancing natural drought conditions by intercepting part of the rainfall before it reaches the ground. A wide range of experiments have been set up in natural ecosystem (Hoover et al., 2018) and already brought results on the effect of pluriannual rainfall reduction and soil water content on plant ecology (Pérez-Ramos et al., 2010). Such design can be applied to adult coffee plants during consecutive production cycles to assess yield under drought conditions.

In the present study, we assessed the effects of decreased soil water availability on growth, yield, and physiology on 5 genotypes of *C. arabica* by using a rainfall reduction experiment in the Northwest of Vietnam. To our knowledge, this study is the first one drawing a link between environmental conditions, plant physiology and yield in *C. arabica* adult trees planted in the field. Our study aimed to answer the following questions: (i) to what extent does soil water deficit affect the agronomical and physiological responses of the various coffee genotypes? (ii) to what extent do physiological responses along the day and across the year explain variations in the agronomic performances of various genotypes? and (iii) how does soil water deficit impact the genotypic response during periods of high evaporative demand?

We make the hypothesis that F1-hybrids will show signs of drought tolerance and display a homeostasis of their vegetative growth and yield in conditions of soil water deficit. We expect that these genotypes will develop their root systems as an adaptation mechanism to maintaining water intake and avoiding loss of turgor and low leaf water potential. Furthermore, we expect that these genotypes will maintain a high stomatal conductance and relatively high photosynthesis under conditions of high evaporative demand in the middle of the day and during the dry season. In comparison, we make the hypothesis that pure lines who were mainly selected for their high yield under optimal conditions and their resistance to leaf rust will be sensitive to drought. We hypothesize that these pure lines will experience a decrease in vegetative and reproductive growth under conditions of soil water deficit and high evaporative demand resulting from reduction in their photosynthesis and stomatal conductance.

## 2 Materials and methods

The experimental design detailed in this article aimed at documenting the vegetative growth and production of 5 C*. arabica* genotypes under a long-term field rainfall reduction treatment. In addition to the long-term agronomical changes observed, we also investigated physiological responses to short-term changes in evaporative demand. Plant material, spatial design, methods and frequency of measurement of each agronomical and physiological parameters are detailed in the subsequent section of the materials and methods.

### 2.1 Plant material

Five *C. arabica* genotypes were used in the trial: two pure lines (a local Catimor [CAT], used as control, and Marsellesa [MAR]) and three intraspecific F1-hybrids (Starmaya [STA], Centroamericano [CEN] and Mundo Maya [MUN]). Pure lines, CAT and MAR, originated from crosses (female x male) between Timor Hybrid 832/1 x Caturra and Timor Hybrid 832/2 x Villa Sarchi CIFC 971/10, respectively (Marie et al., 2020). The F1-hybrid STA resulted from a cross between the sterile male CIR-SM01 and MAR, and hence can be propagated by seeds through biclonal seed garden (Georget et al., 2019). The F1-hybrids CEN and MUN resulted from a cross between the Sarchimor T5296 x Rume Sudan and Sarchimor T5296 x Ethiopian ET01, respectively. Given their heterozygous genetic structure F1 hybrids have to be multiplied by asexual way.

**Figure 1:**
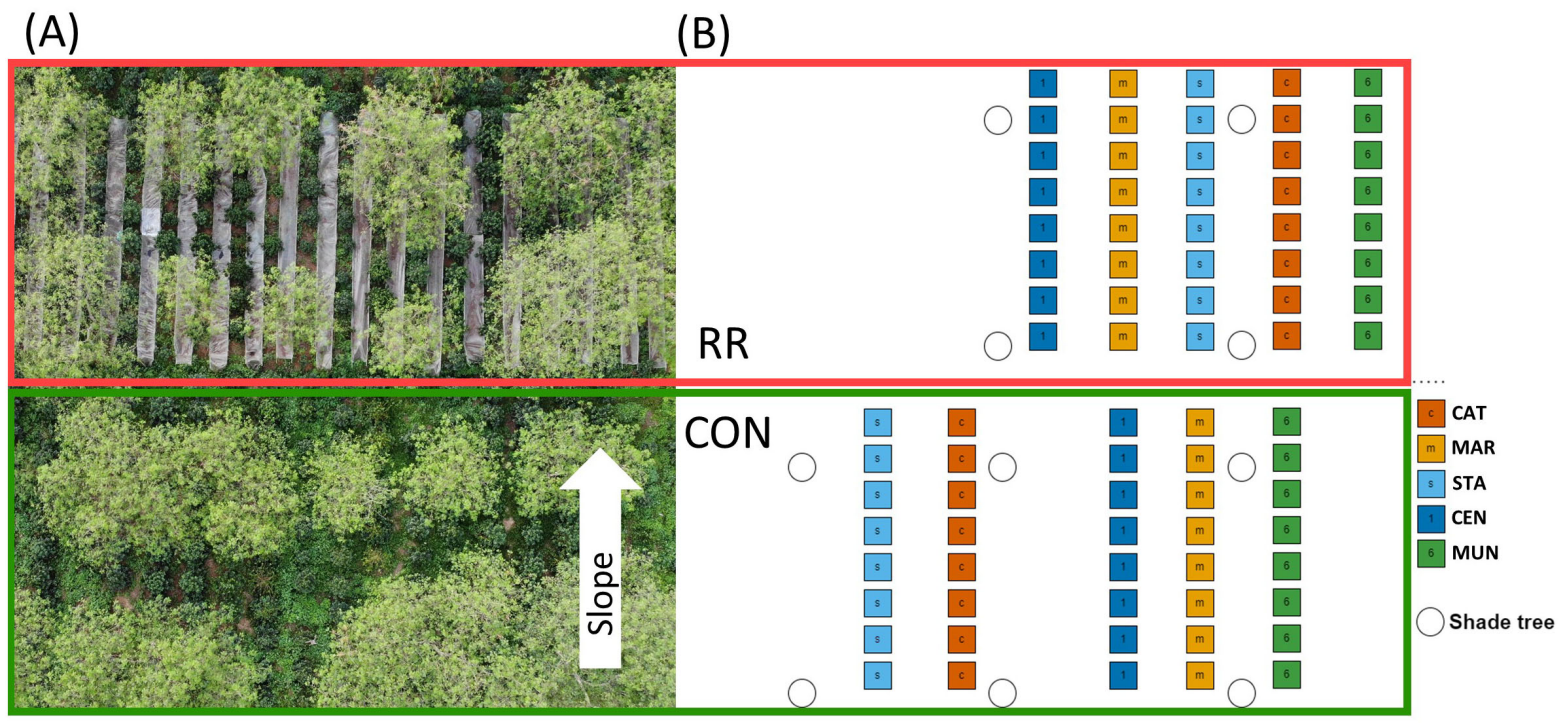
Experimental design of the trial studied in the northwest of Vietnam. **(A)** Flycam picture of part of the trial with the visible shade trees and rainfall reduction system (RR: upper part with vertical plastic bands and lower part: CON for control condition). **(B)** Detailed map of one repetition block of the trial with the 5 studied genotypes (CAT, Catimor; MAR, Marsellesa; STA, Starmaya; CEN, Centroamericano and MUN, Mundo Maya). The white arrow indicates field slope direction.

Seeds of the local CAT were collected in November 2017 from a unique high yielding coffee plant available in the coffee germplasm of NOMAFSI research station (Mai Son district, Son La province, Vietnam). Seeds of MAR and STA were provided by ECOM-Nicaragua within the framework of the H2020 BREEDCAFS European project. All seeds were germinated and grown from November 2017 to April 2018 at the Agriculture Genetics Institute (AGI, Hanoi, Vietnam). The F1-hybrids CEN and MUN were produced by somatic embryogenesis (Etienne et al., 2018) and were sent to Vietnam after reaching the pre-germinated phase for ex vitro plant conversion and initial developmental stages at AGI (from November 2017 to April 2018). Plantlets of MAR, STA, CEN, MUN with a similar development (4-5 leaf pairs) were later transferred in bags to NOMAFSI nursery for a 2-month additional growth period to reach a 30-cm height before being planted in the field trial in July 2018 (during the rainy season). The experimental design of the trial is detailed in the **Figure 1** below.

### 2.2 Location and experimental design

The study was carried out at NOMAFSI research station in Son La province (annual average temperature 21°C, annual rainfall 1500 mm) of Northwest Vietnam at an elevation of 780 m.a.s.l. The trial was initiated in 2018 under AFS with a leguminous tree species, namely *Leuceana leucocephala* planted in 2006. The average shade level during the experiment was estimated with the Licor 6400 to be around 40%.

The trial was a split plot design with 4 repetition blocks (**Figure 1**, design of one block). For phenotyping and harvest measurements, each repetition block was constituted of 80 trees and considered as an independent experimental unit including two water treatments (Control [CON] vs Rainfall reduction [RR]), with a line of 8 plants of each of the 5 genotypes (CAT, MAR, STA, CEN and MUN). Plant spacing was 2 m between rows and 1.5 m within rows. Two lines of CAT genotype were used as “border” separating the blocks and treatments. Treatments were separated by a 50-cm deep trench to eliminate sub-surface water flow between treatments. The RR treatment set up in April 2020 consisted in 15-m long and 1-m wide plastic sheets put between coffee rows (**Figure 1**). A 7% slope allowed the evacuation of the intercepted rainfall to the lower side of the plot and into trenches. Each plastic sheet was raised 40 cm above the ground with steel poles to avoid soil warming. By collecting water in large basins at the lower end of the plastic sheet and comparing it with the rainfall volume measured at a rain gauge, we estimated that the RR system excluded about 35% of the total annual rainfall.

### 2.3 Environmental monitoring

**Figure 2:**
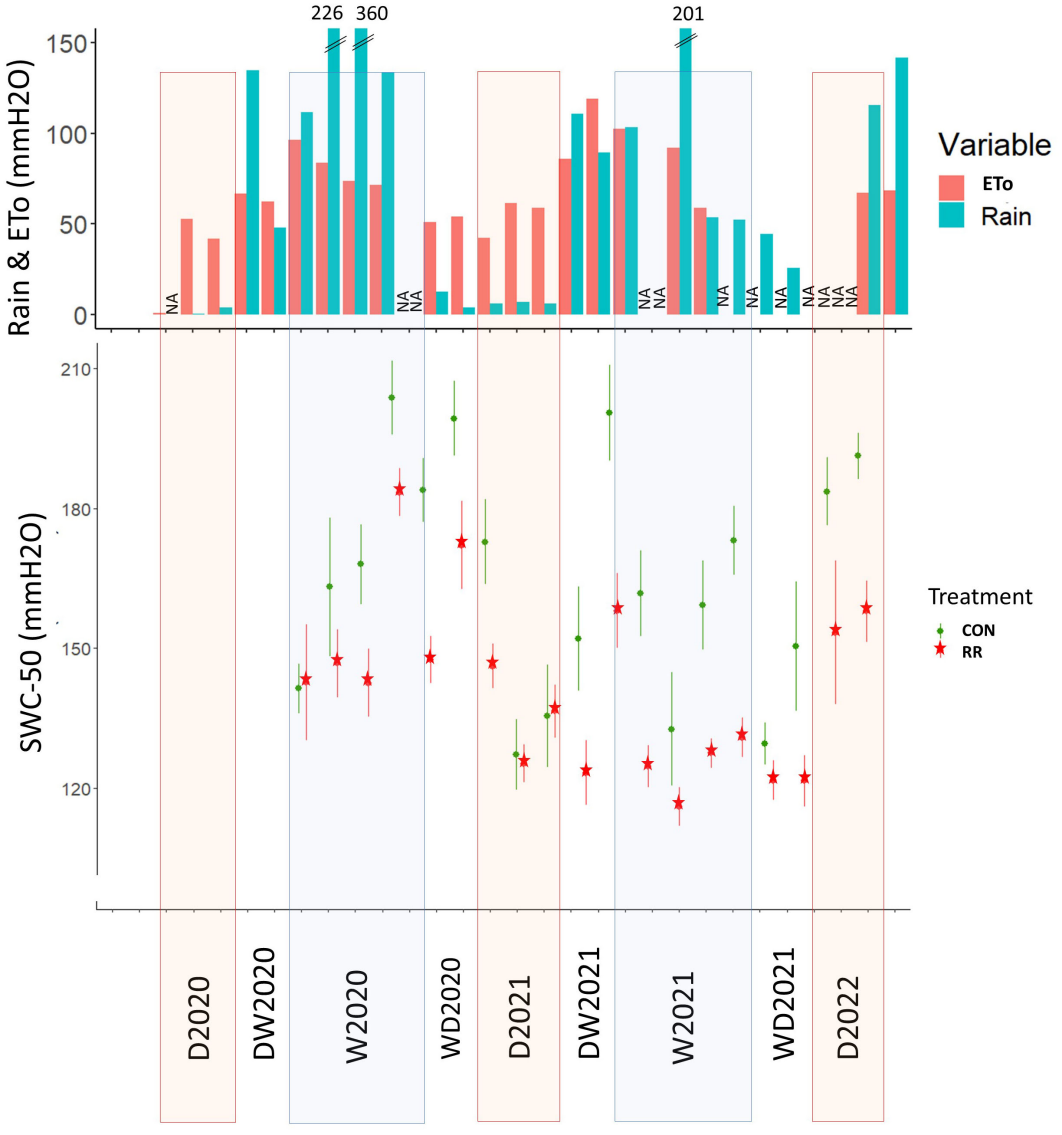
Environmental data across the experiment period. Rainfall and evapotranspiration (Rain & ETo) and soil water content in the first 50 cm (SWC-50). Dry season (D), wet season (W), transition period from dry to wet season (DW) and from wet to dry season (WD), in 2020 (20), 2021 (21) and 2022 (22). NA correspond to data not-available.

Environmental conditions were monitored from January 1st 2020 to April 15th 2022 to cover 3 consecutive dry seasons. Seven environmental variables were monitored: average, minimum and maximum temperatures (Tavg, Tmin, Tmax), average and maximum vapor pressure deficit (VPDavg, VPDmax), soil water content down to 1.5 m, daily rainfall (RF) and potential evapotranspiration (ETo). The air temperature and humidity were recorded every hour using 3 Hygrochron Temperature and Humidity iButtons (DS1923-F5; Embedded data systems, USA). The VPD was calculated with the temperature and humidity following Equation 1 (Allen et al., 1998) below:


(1) $$VPD=\;\frac{H}{100}*\frac{610.7*{10\;}^{\frac{7.5T}{237.3+T}}}{1000}$$

where T is air temperature and H is air humidity. Daily data from the 3 iButtons were used to calculate daily average, minimum and maximum of temperature and VPD as well as standard errors (**Supplementary Figure S1**). Soil water content was measured with a Diviner 2000 probe (Sentek Technologies, Australia) every 10 cm between the soil surface and down to 1.5-m depth in 4 access tubes per treatment. Measurements were taken every two weeks. Data of the four tubes on a same treatment were averaged to get an overall soil water content of that treatment. Soil water content between 0 and 50 cm were summed up to obtain an average soil water content of the first 50 cm (SWC-50), the depth at which 65% of coffee roots are concentrated (Padovan et al., 2015; Rigal et al., 2019). Hourly RF data were collected with a weather station Vantage Pro 2 (Davis Instruments, USA) and summed by day to obtain daily rainfall. Evapotranspiration under AFS was calculated using MeTo R package and the FAO Penman-Monteith equation (Allen et al., 1998) combining data from the weather station and iButtons. Missed RF and ETo data (due to weather station technical issues, i.e. battery, wire disconnection) in **Figure 2** were indicated as not available (NA).

### 2.4 Phenotyping measurements and harvest

Phenotyping measurements, namely the longest plagiotropic branch length (BL) and the tree height (H), were carried out in May 2019, 2020, 2021 on 4 plants per genotype and treatment in each of the 4 repetition blocks. We measured the plagiotropic branch length with a 5-m ruler, from its base connected to the trunk to its tip. Similarly, we measured the height starting from the bottom of the coffee tree to its upper tip. Six plants per genotype and treatment in each of the 4 repetition blocks were selected randomly, harvested and weighted individually at harvest peak between October and December 2021 and 2022, when coffee trees were respectively 3 and 4 years old. The yields of 2021 and 2022 were summed up to obtain a cumulative yield (cumYield), accounting for coffee biennial bearing. There were 32 trees per genotype, 80 trees per treatment and therefore 16 trees selected randomly per genotype-treatment for plant phenotyping. For the harvest, there were 48 trees per genotype, 120 trees per treatment and therefore 24 trees selected randomly per genotype-treatment. For both the phenotyping and harvest data, the data were averaged by block before doing the ANOVA.

### 2.5 Fine root distribution

At the end of the experiment (April 2022), four trenches of 1-m length and depth were dug (15-cm away from the coffee trunks and within the rows) for each genotype and treatment to count the coffee root impacts corresponding to any exposed root intersection in the soil profile opened by the trench (Padovan et al., 2015). To do so, the one square meter soil profile was divided into a grid with square sections of 400 cm^2^ to ease measurement. Root impacts were counted within each 400 cm^2^. The root impacts were summed within each profile to compare the total number of root impacts per m^2^ between genotypes and treatments. In total, there were 40 tree root systems measured, 8 per genotype, 20 per treatment, therefore 4 tree root systems per genotype – treatment.

### 2.6 Physiological measurements

#### 2.6.1 Whole-tree sapflow

In 2020, 5 trees per genotype and treatment were selected for sapflow measurements in the last repetition block of the experiment. Only trees from this last repetition block were reachable by the electrical cable connecting whole-tree sapflow probes and dataloggers. Whole tree sapflow was measured with the Granier probe method (Granier, 1987; Sarmiento-Soler et al., 2019). Since coffee trees were still young, the length of the Granier probes was reduced to 5 mm instead of the commonly used 20 mm probes. The probes were inserted in the coffee trunk at 15 cm above the ground, and connected to an AM16/32 multiplexer (Campbell Scientific Logan UT, USA) and a datalogger CR10X (Campbell Scientific Logan UT, USA), itself connected to a 12 V car battery. The probes were also connected to the grid through two converters, the first one lowering the power to 12 V and the second one to 1.7 V. This voltage was suitable to supply a 1.40 mA current to the warm probe. The datalogger measured the voltage difference in the Granier-type probes every minute and averaged the values every half hour from January 2020 to April 2022. The sapflow system was checked and data was retrieved once a week (except in July and August 2022).

Collected voltage data were cleaned and transformed. Voltage differences were first cleaned based on graphic visual inspection and converted into a sapflow density using the TRACC package (Ward et al., 2017) in R. Zero-flow and voltage max used to calculate the voltage differential and sapflow density were defined using the Oishi baseliner method (Oishi et al., 2022). Voltage max was determined at the time interval of at least 1.5 h at which VPD reached a value of<0.1 as recommended in previous studies to correct for reverse and nocturnal sap movement (Forster 2014; Rabbel et al., 2016; Ward et al., 2017). The experimental system used to measure whole-tree sapflow (CR10X, AM16/32, Granier probes) was calibrated in a greenhouse with two 4-year-old coffee trees monitored with load cell scales which collected weight loss data every hour for 72h. Sapflow data measured with the field and greenhouse systems were correlated with a simple linear regression (R^2 ^= 0.91) later used to correct sapflow data measured on the field. Sapflow density (expressed in g m^-2^ s^-1^) were converted to sapflow per leaf area (in mol m^-2^ LA^-1^ s^-1^) with the following Equation 2:


(2) SapLA= SapD∗SA /  LA / 0.055

where SapLA, SapD, SA and LA corresponded to sapflow per leaf area, sapflow density and sapwood area, and leaf area, respectively, and 0.055 is the constant used to convert g H_2_O to mol H_2_O.

#### 2.6.2 Photosynthesis and stomatal conductance

Measurements of photosynthesis and stomatal conductance were conducted from November 2020 to April 2022 using a Licor 6400 (LICOR, Biosciences, Lincoln, NE, USA) on the same trees in the last repetition block of the experiment which were also used for whole-tree sapflow measurements. We took measurements during the dry season to better highlight potential drought adaptation of each genotype. Photosynthesis (*P_n_*) and stomatal conductance (*g_s_*) were measured 1 to 3 days per month to have data across the dry season and transition seasons. Due to the time necessary to take the measurement, only one tree was measured per genotype per treatment per day (hence, 10 trees measured in total each day), however a different tree was randomly selected and measured each day to consider the variability within treatment. Photosynthesis and stomatal conductance were measured on three leaves per tree. A part of the leaf blade was put inside the Licor 6400 chamber, and measurements were taken after 1 min once the readings of *P_n_* and *g_s_* stabilized. Three measurements were taken per leaf at a 5-second interval and later averaged to have only one value per leaf. Measurements were done at three times of the day corresponding to 7:00-8:30 AM (7AM), 11:00-12:30 AM (11AM) and 2:00-3:30 PM (2PM) reproducing the protocol of a past study (Franck and Vaast, 2009). All measurements were taken in natural and fluctuating conditions of irradiance, temperature, and VPD. All dates of measurements can be found in **Supplementary Materials** (**Supplementary Table S1**). In total, there were 1,440 data points collected (16 days * 3 times of day * 3 leaves per tree * 5 genotypes * 2 treatments).

#### 2.6.3 Pre-dawn leaf water potential

Pre-dawn leaf water potential (*Ψ_PD_*) measurements were taken monthly during the dry season (from November 2020 to April 2021) with a Schölander bomb (PMS Instrument, Albany, OR, USA) at night (from 2:00 to 5:00 AM.). The measurement was conducted on four trees per genotype and treatment, with one leaf per tree (hence, 40 trees measured in total each night). The measurements of the three leaves were later averaged to have one data per tree. The Schölander bomb was located at the edge of the plantation, a few meters from the trees, so that measurements were taken very quickly after leaf harvesting. The leaf was cut by a first operator and put under pressure in the Schölander bomb by a second operator for measurement within 2 min after excision. In case the leaf was covered with dew, it was dried out with a tissue before entering the chamber, therefore the chamber was kept at ambient conditions. Measurements were taken quickly (in less than 2 min) at night when stomata were closed to prevent any water loss. Trees used to measure pre-dawn leaf water potential were the same trees used for *P_n_*, *g_s_* and whole-tree sapflow measurements and therefore they were all located in the same repetition block. The area of each leaf was also recorded using LeafByte mobile application (v1.3.0) (Getman-Pickering et al., 2020) and used together with number of leaves per tree to estimate whole tree leaf area.

### 2.7 Data cleaning and filtering

All negative values of sapflow were excluded. All values above the threshold value (T) based on interquartile range (Equation 3) were also excluded from the analysis (Owuor et al., 2022).


(3) T= Q3 + 2 ∗ (Q3 – Q1)

with Q1 and Q3 corresponding to the first and third quartile, respectively.

We also excluded data belonging to trees and days for which there were missing data points (fewer than 48 data points per tree-day). Similarly, the negative *P_n_* and *g_s_* data were excluded from the analysis, as well as the outliers above the defined threshold (Equation 3). In addition, these data were only available on 16 days and at three-time intervals each (**Supplementary Table S1**). Therefore, for comparison purposes, we filtered whole-tree sapflow data at the same day and time intervals as previously described for Licor measurements. The sixteen days were representatives of dry, wet seasons and the transition period between these two seasonal extremes. After filtering sapflow data, we had a total of 1440 data points (16 days * 3 times of day * 3 repetitions * 5 genotypes * 2 treatments).

### 2.8 Statistical analyses

For phenotypic measurements, root count and cumulated yield data, we used ANOVA and *post-hoc* Tukey test to assess the significance of the differences between genotypes within a treatment as well as between treatments within a genotype. Normal distribution, independence of data and homogeneity of variances within each group were respected. Interactions between the genotype and treatment fixed effects were considered as well. Block effect for height, branch length and cumulated yield per tree, as well as tree effect for root density were treated as random. For *P_n_*, *g_s_* and whole tree sapflow, ANOVAs were carried out to highlight the effect of the treatment, genotype, and time of day, season, and their interactions. ANOVA assumptions were verified. Tree effect was treated as random. *Post-hoc* Tukey test were carried out to highlight significant differences between genotypes at each time of the day. The variation of *P_n_*, *g_s_* and sapflow across seasons was graphed using a day average of the three data points collected at the time intervals defined previously. Finally, we built simple linear models of whole tree sapflow, *P_n_* and *g_s_* in function of ETo for each genotype. All statistical analyses were performed on R Version 4.2.2.5 (R Core Team, 2022). Data were visualized with the R packages ggplot2 and ggpubr. A **Supplementary Table** (**Supplementary Table S2**) summarizes the nature, method, period and frequency of measurements of all variables in this study.

## 3 Results

### 3.1 Environment and soil water deficit

The environmental conditions in the study site displayed a high seasonality with a cold and dry season from November to March (Tavg = 12°C), and a hot and wet season from April to September (Tavg = 23°C) (**Supplementary Figure S1**). VPD peaked in April and May, with a higher peak in 2021 (VPDmax = 4.2 kPa) than in 2020 (VPDmax = 3.2 kPa). Total annual potential evapotranspiration (ETo) amounted to 716 mm in 2020 and 861 mm in 2021. Total annual rainfall in 2020 and 2021 were 1194 mm and 1016 mm of which 95% and 83% respectively happened between April and September (**Figure 2**). The dry season (from January to March) was defined when monthly rainfall was lower than monthly ETo. Despite earlier rainfall in March 2022, we classified the month of March 2022 as part of the dry season in order to be consistent with 2020 and 2021. From the onset of RR treatment in April 2020, an average soil water deficit of 14% was observed in the top 50 cm of the soil compared to the control treatment (**Figure 2**).

### 3.2 Effect of genotype and rainfall reduction on the agronomical and physiological performances of the 5 coffee genotypes

**Figure 3:**
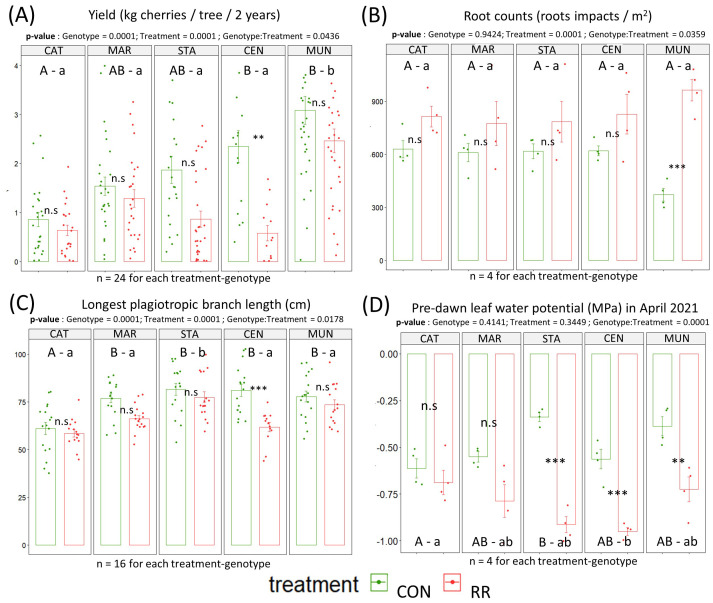
Performances of the *C*. *arabica* genotypes (Catimor [CAT], Marsellesa [MAR], Starmaya [STA], Centroamericano [CEN] and Mundo Maya [MUN]) tested under rainfall reduction (RR) or control (CON) conditions. The barplots presented the cumulated yield of 2020 & 2021 **(A)**, root counts **(B)**, longest plagiotropic branch length **(C)**, and ΨPD (pre-dawn leaf water potentials) in April 2021 **(D)**. Each dot is a data collected on one tree. Vertical bars in the bar plots are standard errors. Capital letters above isobars show significant differences between genotypes under CON, while lowercase letters show significant differences between genotype under RR treatment. Same letters mean no significant differences between genotypes. P-values show significant difference between the CON and RR treatments for a given genotype. Significance codes: 0 ***, 0.001 **, 0.01 *, 0.05 n.s, 1. n.s stands for non-significant.

Even though, water potentials were lower during the dry (D) season than during the wet (W) season, the mild dry season always maintained Ψ_PD_ above –1 MPa. Because of weak water stress, pre-dawn leaf water potentials ranged from -0.26 MPa to -1.0 MPa and were not affected by the genotype and treatment except at the end of the dry season in April 2021 (**Supplementary Table S3**). At this time, under CON condition, CAT had a significantly lower Ψ_PD_ (-0.6 MPa) than STA (-0.3 MPa); and in the rainfall reduction treatment, CAT had a significantly higher Ψ_PD_ (-0.7 MPa) than CEN (-0.9 MPa). In addition, F1-hybrids STA, CEN and MUN showed a significantly higher leaf water potential in CON compared to RR treatments while CAT and MAR pure line varieties did not display any effect of the treatment on this parameter (**Figure 3**). *P_n_*, *g_s_* and sapflow were similar in both CON and RR treatments with no significant differences throughout the experimental period.

**Figure 4:**
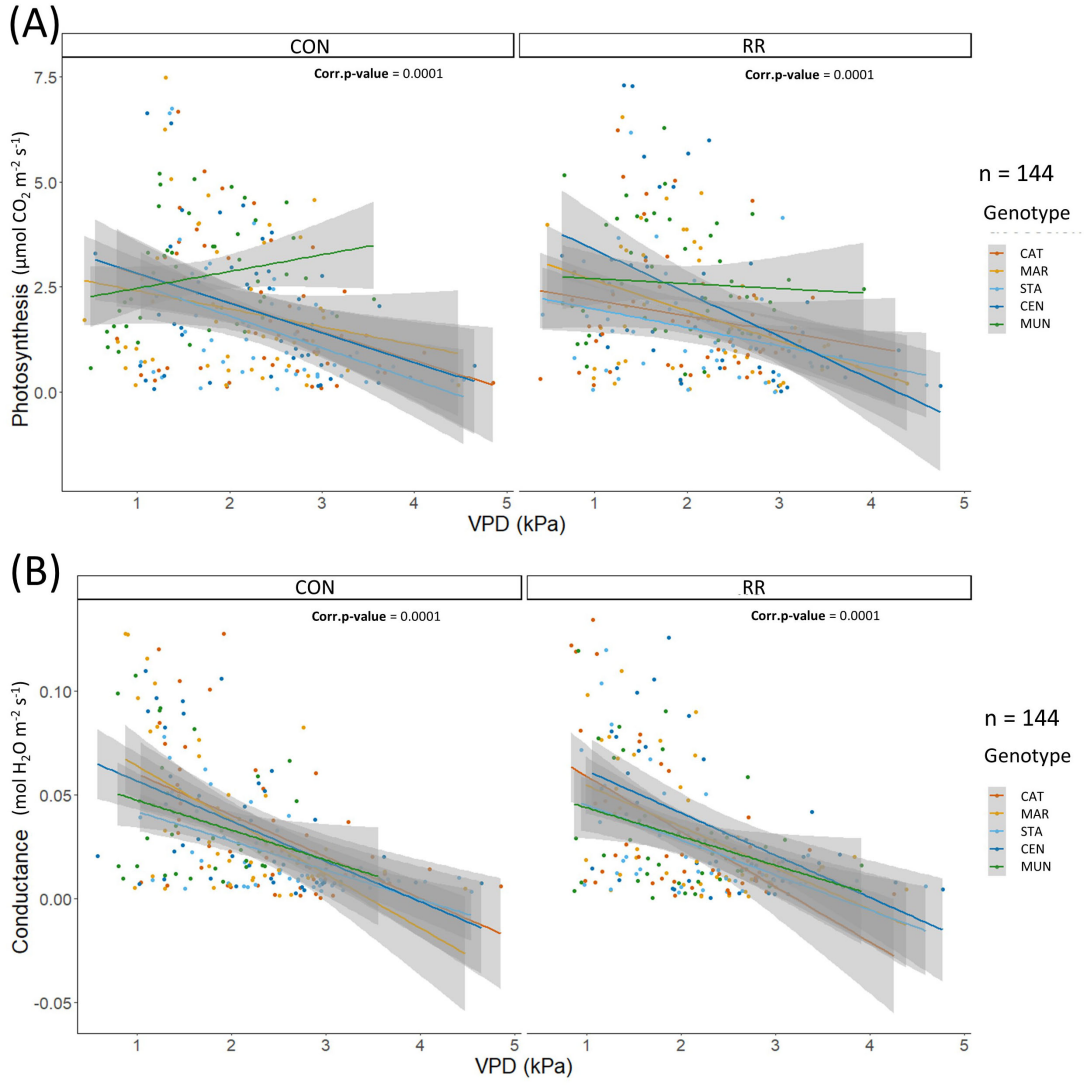
**(A)** Photosynthesis (expressed in µmol CO_2_ m^-2^ LA s^-1^), **(B)** conductance (expressed in mol H_2_O m^-2^ LA s^-1^) in function of hourly VPD in kPa for the rain-fed (CON) vs rain reduction (RR) treatments of the 5 genotypes (CAT: Catimor, MAR: Marsellesa, STA: Starmaya, CEN: Centroamericano, MUN: Mundo Maya). Colored lines are simple linear models and grey areas are confidence intervals.

Under CON treatment, CEN and MUN showed significantly higher cumulated yield over 2 years than CAT, MAR and STA genotypes. Under RR condition, the MUN F1-hybrid had a significantly higher cumulated yield than all other genotypes (**Figure 3**). With respect to genotype response to drought treatments, no significant change in cumulated yield was observed between treatments for most of genotypes, except for CEN which showed a significant decrease in cumulated yield under RR treatment compared to CON condition. Interestingly, the CEN F1-hybrid, which yielded better than CAT genotype under CON, had its yield reduced by more than half under RR treatment resulting in a low yield equivalent to that of CAT genotype. Under both treatments, no differences were observed regarding tree height across genotypes except for STA hybrid which was significantly the tallest genotype under CON and also taller than CAT and CEN genotypes under RR treatment (**Supplementary Figure S2**). Similarly, we observed no significant differences in branch length across genotypes within treatments, except for CAT which had significantly the shortest branch in CON conditions, and STA which had the longest branch under RR condition (**Figure 3**). Within genotypes, there were no significant effects of the RR treatment on height and branch length, except for CEN hybrid whose branch length was shorter in the RR treatment compared to CON condition (**Figure 3**). MAR and STA had the highest total tree leaf area in CON and RR treatments reaching 10 m^-2^ while CAT had the lowest in both treatments reaching only 5 m^-2^ (**Supplementary Figure S2C**). Total tree leaf growth across the time of the experiment showed a consistent lower leaf area in CAT (**Figure 4**).

### 3.3 Contrasted physiological response of 5 coffee genotypes to seasons and time of day

**Figure 5:**
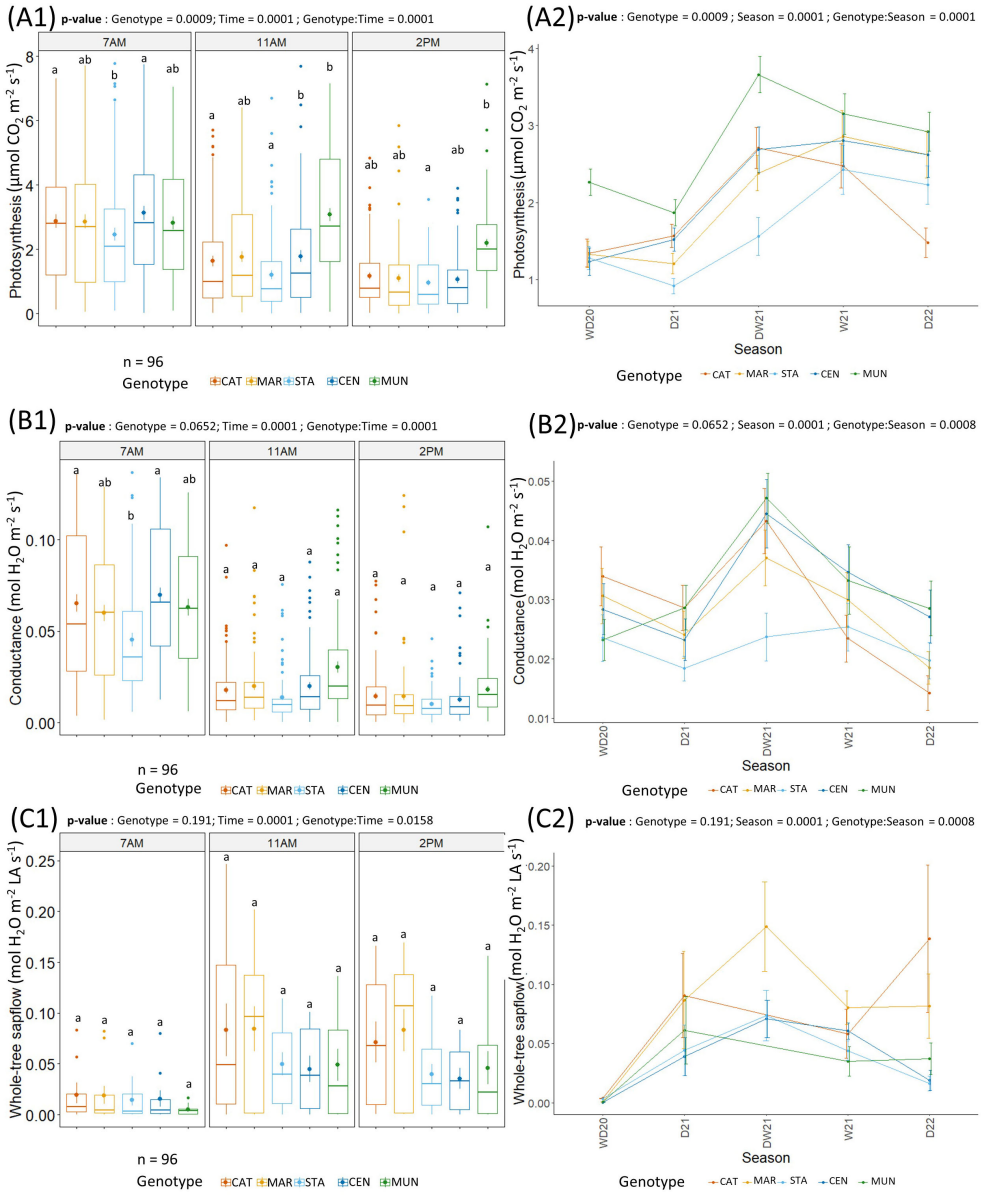
**(A)** Photosynthesis (µmol CO_2_ m^-2^ LA s^-1^), **(B)** stomatal conductance (mol H_2_O m^-2^ LA s^-1^) and **(C)** Whole-tree sapflow (mol H_2_O m^-2^ LA s^-1^) of the 5 genotypes (CAT: Catimor, MAR: Marsellesa, STA: Starmaya, CEN: Centroamericano, MUN: Mundo Maya). Boxplots show a grand average of the photosynthesis **(A1)**, conductance **(B1)** and whole-tree sapflow **(C1)** per genotype and time of day. Bold dots and the vertical line in the boxplot are the average and the standard error, respectively. Dots outside the boxplot are outliers. Lower case letters indicate Tukey-test significance with 95% confidence, genotypes with a same lower-case letter are not significantly different. Line graphs show the variation of photosynthesis **(A2)**, conductance **(B2)** and whole-tree sapflow **(C2)** per genotype and time of day across seasons. Dry (D), wet seasons (W), transition periods (DW, WD) in 2020 (20), 2021 (21), and 2022 (22).

No significant differences were observed between treatments on coffee physiology (**Supplementary Table S3**). Therefore, physiological measurements were pooled across treatments before studying short-term temporal variations that permitted observing an effect of genotype with time of day and seasons (**Figure 5**). With pooled data, time of the day, seasons and their interactions with genotype significantly influenced *P_n_*, *g_s_*, and sapflow (**Supplementary Table S3**). Values of *P_n_* and *g_s_* were rather low along the whole experimental period spanning from 1 to 4 µmol CO_2_ m^-2^ s^-1^ for *P_n_* and 0.01 to 0.05 mol H_2_O m^-2^ s^-1^ for *g_s_*. Both *P_n_* and *g_s_* were at their highest at 7 AM and decreased at 11 AM to be their lowest at 2 PM. Furthermore, these two parameters were higher during the transition of dry to wet season (DW21) than during the dry season (D21, D22), except at 2 PM when all values were low in all seasons (**Figure 5**). All genotypes had a similar *g_s_* at 11 AM and 2 PM. Nonetheless, CAT and CEN genotypes had a significantly higher *g_s_* and *P_n_* than STA hybrid at 7 AM. Moreover, the CEN and MUN hybrids had a higher *P_n_* than CAT and STA genotypes at 11 AM, and MUN had a higher *P_n_* than STA at 2 PM.

For all genotypes, whole-tree sapflow was the lowest at 7 AM and increased at 11 AM and 2 PM. It was higher in the dry season (~0.10 mol H_2_O m^-2^ LA s^-1^) than in the wet season (~0.06 mol H_2_O m^-2^ LA s^-1^). No significant genotype effect was observed for the whole-tree sapflow.

### 3.4 Contrasted physiological responses of coffee genotypes to ETo and soil water deficit

**Figure 6:**
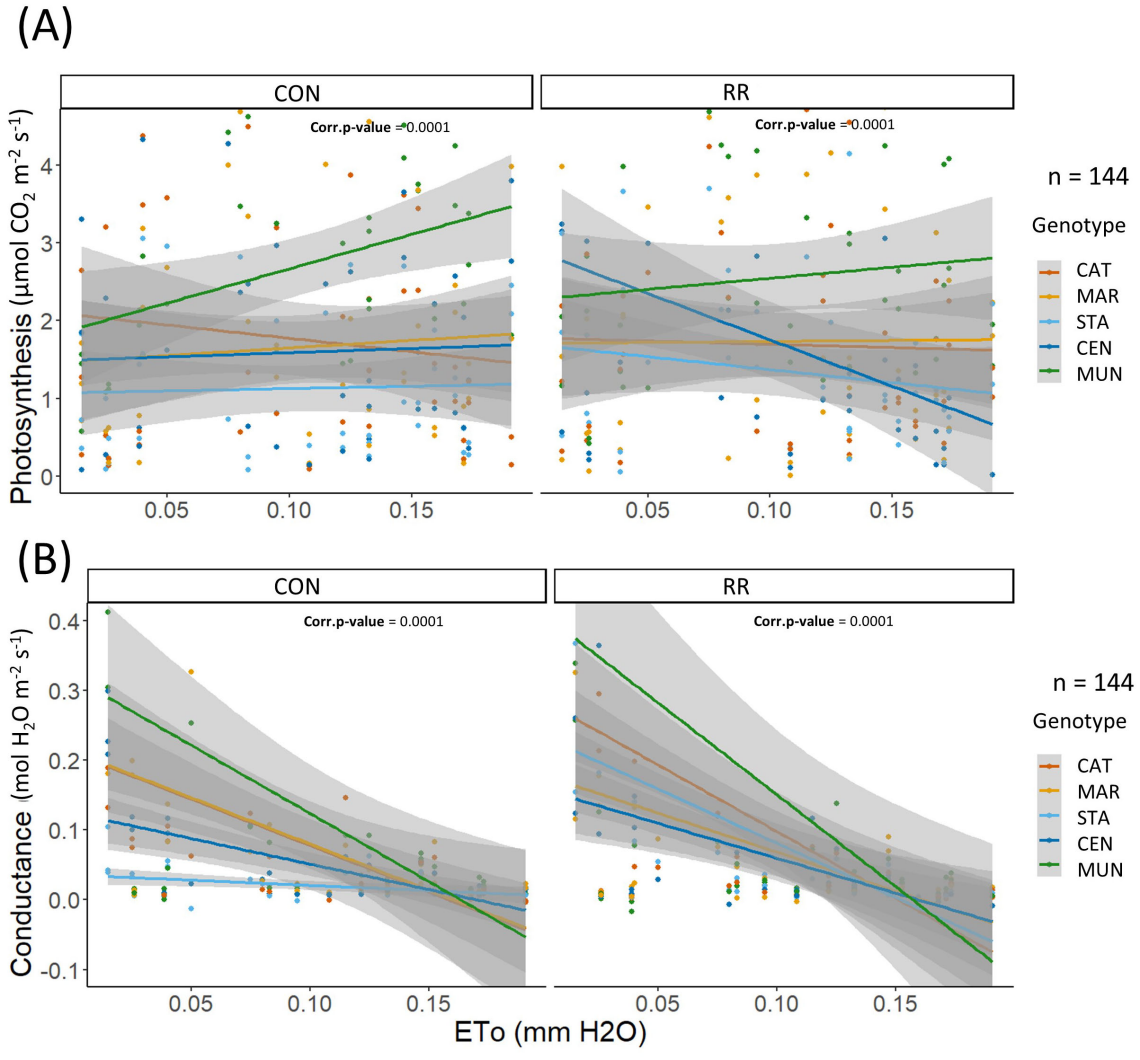
**(A)** Photosynthesis (expressed in µmol CO_2_ m^-2^ LA s^-1^), **(B)** conductance (expressed in mol H_2_O m^-2^ LA s^-1^) in function of hourly ETo in mm for the rain-fed (CON) vs rain reduction (RR) treatments of the 5 genotypes (CAT: Catimor, MAR: Marsellesa, STA: Starmaya, CEN: Centroamericano, MUN: Mundo Maya). Colored lines are simple linear models and grey areas are confidence intervals.

The differences in *P_n_* and *g_s_* among genotypes along the day and across seasons were related to diurnal and seasonal changes in environmental conditions, more specifically changes in evaporative demand (ETo). From simple linear models of these two parameters in function of ETo, we observed that *P_n_* was similar among genotypes along the ETo gradient (0 - 0.2 mm H_2_O h^-1^) under CON condition except for MUN hybrid which had a higher *P_n_* (above 0.1 mm H_2_O h^-1^) than other genotypes at high ETo. Moreover, under CON conditions, *g_s_* was similar among genotypes at high ETo (0.1 – 0.2 mm H_2_O h^-1^), but different at low ETo (0 – 0.1 mm H_2_O h^-1^), with STA hybrid having the lowest *g_s_* while MUN had the highest (**Figure 6**). Under RR treatment, all genotypes maintained a constant *P_n_* along the ETo gradient except CEN hybrid which reduced its photosynthesis, indicating a potential drought sensibility of this hybrid under RR treatment (**Figure 6A**). Responses of *P_n_* and *g_s_* to VPD were similar across genotypes, except for MUN hybrid that had a higher *P_n_* compared to other genotypes at high VPD under the CON condition (**Supplementary Figure S3**). Sapflow stayed constant or increased with higher ETo and VPD, and genotype differences were observed only under CON treatment. Under this condition, MAR had a higher and CAT a lower sapflow than other genotypes in ETo range of 3-4 mm H_2_O/day and VPD range of 0.5-1.5 kPa. For all three physiological variables (*P_n_*, *g_s_* and sapflow), differences among genotypes were observed only under CON treatment indicating a possible absence of the genotype effect through the combined stress of evaporative demand and soil water deficit.

## 4 Discussion

### 4.1 Climate conditions and drought stress

The climatic conditions at the experimental site were representative of the Northern frontiers of coffee plantations in Asia (Cai et al., 2007; Rigal et al., 2020), with high seasonality, low temperature in winter, high potential evapotranspiration (ETo), and high vapor pressure deficit (VPD). To our knowledge, the conditions found at the edge of the coffee belt are similar to scenarios with +2°C temperature increase as predicted by models in Kenya, Peru and Mexico (Pham et al., 2019; Kath et al., 2022), and can inform on the potential impacts of climate change in these areas. Moreover, this study artificially created a long-term rainfall deficit similar to those predicted by various climate change scenarios in certain coffee producing regions of the inter-tropical belt (Bunn et al., 2015; Ovalle-Rivera et al., 2015; Magrach and Ghazoul, 2015). Despite the mild dry period, water potentials in April 2021 were lower in the RR treatment highlighting the efficiency of the rain-out shelter and the effect of the 14% reduction of soil water.

We suspect that extreme environmental conditions combined with low light and high daily variation of temperature (10°C - 30°C) contributed to the low photosynthetic activities and stomatal conductance observed in our study. Moreover, the measurement periods of the physiological variables (i.e. Novembre – June) during the coldest and driest time of the year as well as measurement times at sunrise (7 AM), and at times of high temperature and VPD (11 AM and 2 PM) could explain the low photosynthetic activity and conductance observed in our study. A study in China, using a Licor-6400XT in December and May in a coffee system intercropped with banana found similar ranges of 2.5 - 3.3 µmol CO_2_ m^-2^ s^-1^ for *P_n_* and 0.25 - 0.35 mol H_2_O m^-2^ s^-1^ for *g_s_* (Hao et al., 2022). In comparison, other studies have reported higher values i.e. *g_s_* = 0.1 - 0.6 mol H_2_O m^-2^ s^-1^ and *P_n_* up to 10 µmol CO_2_ m^-2^ s^-1^ (Franck and Vaast, 2009) and averaged values of *P_n_* of 7.2 - 8.3 μmol m^-2^ s^-1^ with maximum stomatal conductance of 0.11 - 0.15 mol H_2_O m^-2^ s^-1^ and minimum values of 0.01 - 0.02 mol H_2_O m^-2^ s^-1^ (DaMatta et al., 2007) or *P_n_* = 5.9 - 8.8 μmol m^-2^ s^-1^, *g_s_* = 0.060 - 0.146 mol H_2_O m^-2^ s^-1^ (Batista et al., 2012). All these studies show the possible range of conductance and photosynthesis values for coffee; and confirm that our values are correct and unbiased.

### 4.2 Drought tolerance in pure lines and F1-hybrids

Using yield as the main indicator for drought tolerance, our study identified four drought tolerant genotypes (and one drought sensitive genotype). Although Arabica intraspecific hybrids have largely shown to be more vigorous and productive than conventional American pure lines both in full sun and agroforestry systems (Bertrand et al., 2005, Bertrand and Lara-Estrada, 2011) and in different environments (Marie et al., 2020), no detailed information is available to date on their tolerance to drought stress. In studies carried out in Nicaragua, the yield of hybrids including STA was significantly higher than the Caturra and MAR pure line genotypes (Georget et al., 2019; Marie et al., 2020). Some characteristics common to all hybrids, such as vigor, productivity and homeostasis in the face of contrasted light regimes, have already been demonstrated (Bertrand et al., 2005, Bertrand and Lara-Estrada, 2011). The initial information, provided in this study, indicates that this will not be the case for the adaptation to drought stress, which varies considerably from one hybrid to another, probably because this trait is inherited from one of the progenitors. We showed for the first time that one F1 hybrid (MUN) maintained high yield and outperformed the local variety CAT in control condition and all genotypes in water limiting conditions hence presenting tolerance to low soil water availability. Our results indicate that it is possible to find highly tolerant hybrids and hence explain why ongoing Arabica breeding programs are selecting for this trait by applying phenotyping at an early stage for drought tolerance (Breitler et al., 2022). Unlike other genotypes, the CEN hybrid was clearly affected by the reduced soil water availability as it considerably reduced its branch length and cumulative yield, suggesting a drought sensitivity of this genotype, which has never been documented before. It should be mentioned that a study in Costa Rica has already shown a higher sensitivity of CEN to an excess of rainfall compared to the well-known Caturra cultivar (Pappo et al., 2021). The CAT pure line, most widely grown throughout Vietnam shows low production compared with the new genotypes tested, but is little affected by the water deficit, revealing its drought tolerance and robustness in the face of climate change.

### 4.3 Ecophysiogical and agronomical mechanisms behind drought tolerance

Among all genotypes, the MUN hybrid was the only one to both display a high cumulative yield and sustain it under low soil water availability. This specificity may be explained by its higher *P_n_* at 11 AM, 2 PM and across seasons as well as its capacity to increase *P_n_* along the ETo gradient suggesting a tolerance to high evaporative demand. This unique physiological response might be linked to MUN number of fine roots that was three times higher in the rainfall reduction treatment compared to the control treatment. On the contrary, CEN had a decreasing *P_n_* along the ETo gradient and its number of fine roots did not change between treatments which caused its yield reduction in RR. This difference between CEN and MUN emphasizes that carbon allocation to fruits or roots is as important as the maintenance of the photosynthetic activity to enable a genotype drought tolerance. At the peak of drought stress, MUN showed less difference in pre-dawn leaf water potentials between treatments than STA and CEN, highlighting a possible link between drought tolerance and homeostasis of turgor. Such a clear relation between morphological, physiological traits and drought tolerance has never been shown before in the *C. arabica* species. In *C. canephora*, drought tolerant clone had higher *g_s_* and *P_n_* than drought sensitive clones in control conditions (Vieira et al., 2013). MUN better performance on this experimental trial in Vietnam confirms farmers’ preference observed in Central America who perceived MUN as highly productive and resistant to pests and diseases (Turreira-García, 2022).

In the search of drought tolerant coffee genotypes, studies have associated drought tolerance to coffee trees with denser canopy architecture, higher stomatal conductance and sapflow (Tausend et al., 2000), higher photosynthesis and leaf sugar content (Martins et al., 2019), higher wood density (Menezes-Silva et al., 2015), higher water potential and deeper rooting depth (Pinheiro et al., 2005). In the mild drought conditions of the present study, all 5 genotypes maintained stomatal conductance, photosynthetic rate and sapflow as already observed in various genotypes of *C. arabica* (Cai et al., 2007; Koutouleas et al., 2023). We also observed an increase of *P_n_* along the ETo gradient in the high producing and drought tolerant genotype (MUN) and a decrease of *P_n_* at high ETo in the drought sensitive genotype (CEN) suggesting that *P_n_* conservation under high evaporative demand is a key physiological trait associated with drought tolerance in *C. arabica*. In annual crops, a growing body of research has been looking at genotypes restricting their transpiration at high evaporative demand as an adaptation to drought (Jyostna Devi et al., 2010; Ryan et al., 2016; Guiguitant et al., 2017; Sinclair et al., 2017; Tamang et al., 2022). In the present study, only maintaining *P_n_* and not transpiration has been identified as an adaptation trait in the 5 C*. arabica* genotypes.

### 4.4 Plant water relations and fine root plasticity as potential traits indicating drought tolerance

While agronomical traits were influenced by soil water deficit, we found that physiological traits were not affected by soil water deficit likely due to the mild drought experienced by the coffee trees during these 2 years. Physiological traits were affected by changes in evaporative demand, in accordance with a previous study reporting that *C. arabica* reduced its photosynthesis and conductance at high VPD (Franck and Vaast, 2009). Moreover, ecological studies in tropical ecosystems have shown that plant water relations are more likely to be affected by high evaporative demand than a limited soil water deficit (Grossiord et al., 2020). This low effect of soil water deficit on plant water relations will highly depend on the magnitude of future water deficit in these ecosystems. Although we could not fully identify the agronomical or physiological mechanisms behind drought tolerance and sensitivity, we observed a significant change in root density in the MUN tolerant genotype which may be linked to better drought acclimation as already documented in annual crops (Schneider and Lynch, 2020; Affortit et al., 2022). The high fine roots might have contributed to MUN lower difference in pre-dawn leaf water potentials between treatments compared to STA and CEN in April 2021 at the peak of drought. This highlights a possible drought tolerance mechanism where the plant fine roots increase to intake more water, preserve turgor and sustain photosynthesis at high ETo and therefore conserve high yield in dry conditions. Such probable adaptation mechanism and agronomical traits are documented in fruit-bearing adult coffee trees for the first time in this study.

## 5 Conclusion

In conclusion, new genotypes have a high yield potential in the northwest of Vietnam. However, drought tolerance and the maintenance of the yield in reduced soil water availability could not be generalized to F1-hybrids as each genotype responded differently to drought stress. Our study identified maintenance of photosynthesis in conditions of high evaporative demand as well as enhanced root density in low soil water availability as adaptation traits associated to drought tolerance for *C. arabica*. Two high yielding genotypes (CEN and MUN) showed a contrasting physiological response to high evaporative demand, and a divergent response in number of fine roots to rain reduction which finally led to a contrasted response to drought tolerance measured by the branch growth and yield. Plant water relations such as maintenance of sapflow and stomatal conductance were not linked to drought tolerance in our experiment. Our results are applicable to shaded systems which are common in smallholder farms throughout the tropical belt. However, the difference observed between genotypes are likely to also be observed in other environments such as full sun systems. Our improved understanding of the physiological and agronomical responses of Arabica pure lines versus F1 hybrids to low soil water availability in agroforestry conditions will enhance coffee breeding and genotype selection for drought tolerance as a climate smart alternative for resilient coffee agroecosystems.

## Data Availability

The raw data supporting the conclusions of this article will be made available by the authors, without undue reservation.
