# Supplementary material for: Contrasted agronomical and physiological responses of five Coffea arabica genotypes under soil water deficit in field conditions
Source: Front Plant Sci. 2024 Oct 8;15:1443900. doi: 10.3389/fpls.2024.1443900 (PMC11500665; doi:10.3389/fpls.2024.1443900)
Supplement: Supplementary file 1 [file DataSheet1.docx]

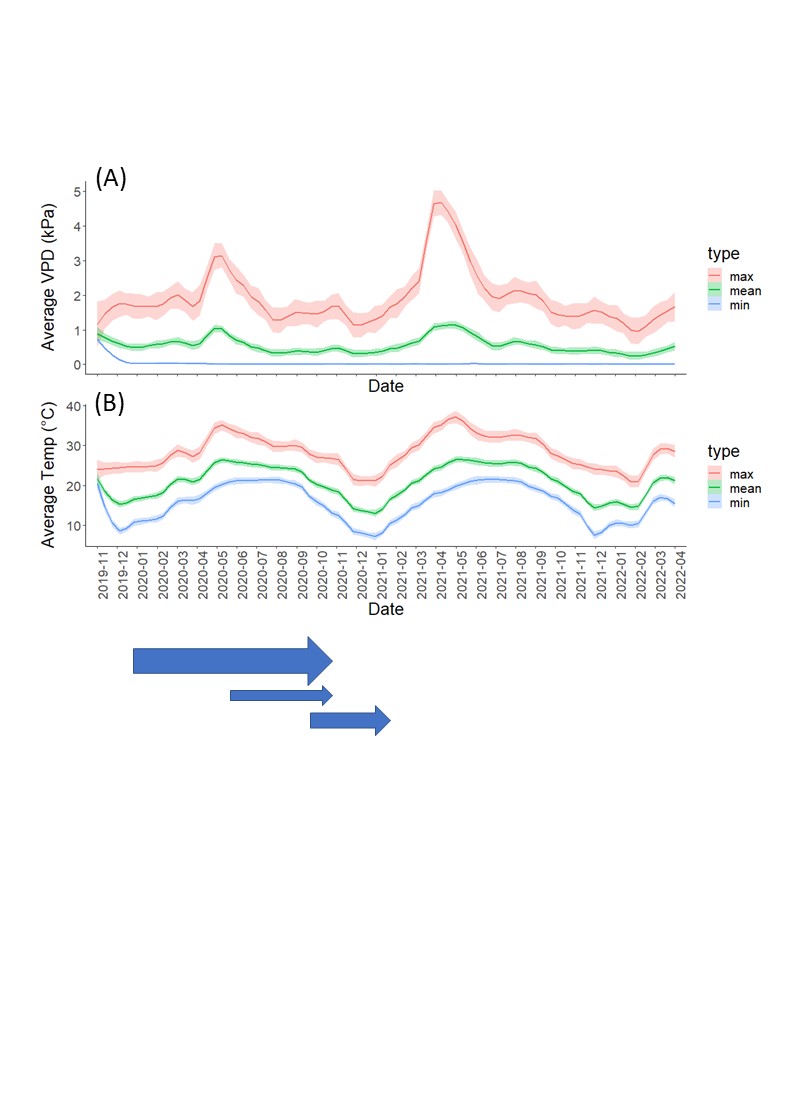


**Supplementary Figure S1.** Vapor pressure deficit (VPD) in kPa (A) and temperature in degree Celsius (B) across the experiment period.


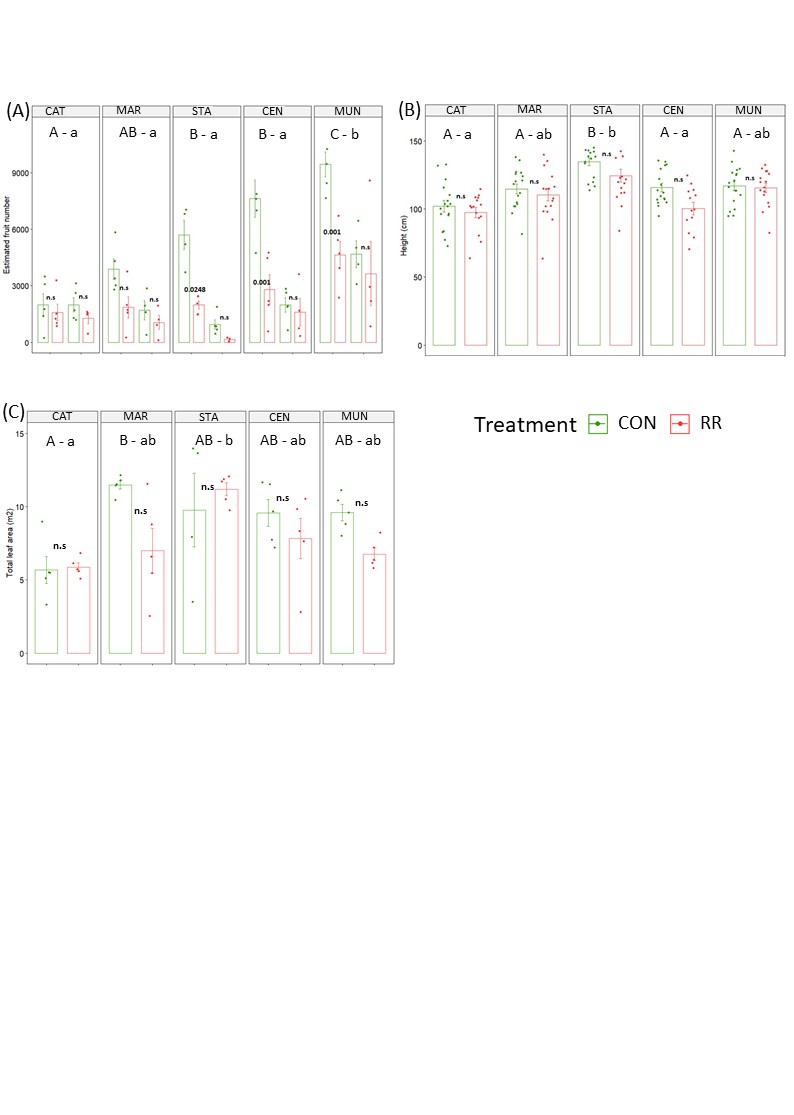


**Supplementary Figure S2.** Performances of the 5 genotypes (CAT: Catimor, MAR: Marsellesa, STA: Starmaya, CEN: Centroamericano, MUN: Mundomaya in water deficit trial (CON: control in green, RR: rain reduction in red) for the estimated fruit number (A), height (B) and total leaf area (C). Capital and small letters show significant differences between genotypes in the control and water supressed treatment, respectively. Genotypes with a same letter are not significantly different. P-value show significant difference between the control and water supressed treatment for a given genotype, and at a given year for the estimated fruit number.


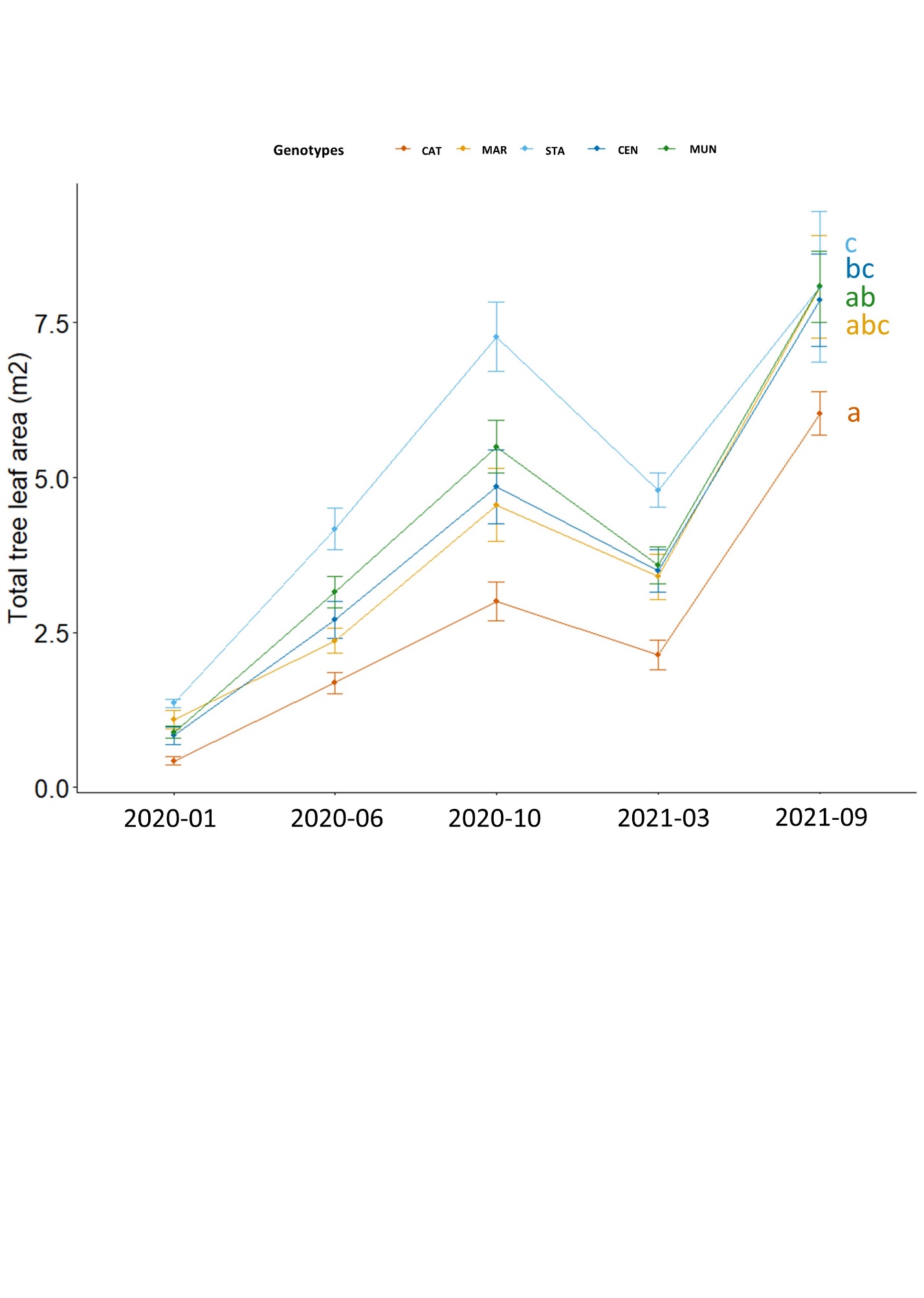


**Supplementary Figure S3.** Total tree leaf area of the 5 genotypes (CAT: Catimor, MAR: Marsellesa, STA: Starmaya, CEN: Centroamericano, MUN: Mundomaya. Small letters show significant differences between genotypes. Genotypes with a same letter are not significantly different.

| **Month** |  | **Water-potential** | **Leaf gas exchange** |
| --- | --- | --- | --- |
| Nov-20 | WD20 | 25/11/2020 | 21/11/2020 |
|  |  |  | 24/11/2020 |
| Dec-20 | WD20 | 15/12/2020 | 24/12/2020 |
|  |  |  | 26/12/2020 |
| Jan-21 | D21 | 06/01/2021 | 02/01/2021 |
| Feb-21 | D21 | 02/02/2021 | 15/02/2021 |
|  |  |  | 21/02/2021 |
| Mar-21 | D21 | 02/03/2021 | 06/03/2021 |
| Apr-21 | DW21 | 02/04/2021 | 31/03/2021 |
| May-21 | DW21 | 20/04/2021 | 01/05/2021 |
|  |  |  | 04/05/2021 |
| Jun-21 | W21 |  | 17/06/2021 |
| Oct-21 | W21 | 26/10/2021 | 06/10/2021 |
|  |  |  | 07/10/2021 |
| Apr-22 | D22 |  | 26/03/2022 |
|  |  |  | 10/04/2022 |

**Supplementary Table S1.** Dates of water potential and leaf gas exchange measurements taken during the experiment.

| Variable | Nature of the quantitative variables | Method of collection | Period | Frequency |
| --- | --- | --- | --- | --- |
| Temperature | Environmental | Hygrochron Temperature and Humidity iButtons (DS1923-F5; Embedded data systems, USA). | 01/01/2020 to 15/04/2022 | Hourly |
| Vapor pressure deficit | Environmental | Hygrochron Temperature and Humidity iButtons (DS1923-F5; Embedded data systems, USA). | 01/01/2020 to 15/04/2022 | Hourly |
| Rainfall | Environmental | Weather station Vantage Pro 2 (Davis Instruments, USA). | 01/01/2020 to 15/04/2022 | Hourly |
| Evapotranspiration | Environmental | Hygrochron Temperature and Humidity iButtons ; Weather station Vantage Pro 2. | 01/01/2020 to 15/04/2022 | Hourly |
| Soil water content | Environmental | Diviner 2000 probe (Sentek Technologies, Australia) every 10 cm between the soil surface and down to 1.5-m. | 01/01/2020 to 15/04/2022 | Bi-weekly |
| Branch length | Phenotyping | Measured with a 5-m ruler, from the base to the tip of the longest branch. | May 2019, 2020, 2021 | Yearly |
| Tree height | Phenotyping | Measured with a 5-m ruler from the bottom to the top of the tree. | May 2019, 2020, 2021 | Yearly |
| Yield | Phenotyping | Harvested and weighted the cherries of each tree. | 2021, 2022 | Yearly |
| Fine root impacts | Phenotyping | Counted root impact in a 1-square meter soil profile. | April 2022 | Once |
| Whole-tree sapflow | Physiological | 5-mm Granier probes connected to a AM16/32 multiplexer and a CR10X datalogger (Campbell Scientific Logan UT, USA). | 01/01/2020 to 15/04/2022 | Half-hourly |
| Photosynthesis | Physiological | Licor 6400 gas exchange chamber (LICOR, Biosciences, Lincoln, NE, USA). | 01/11/2020 - 15/04/2022 | Monthly |
| Stomatal conductance | Physiological | Licor 6400 gas exchange chamber (LICOR, Biosciences, Lincoln, NE, USA). | 01/11/2020 - 15/04/2022 | Monthly |
| Pre-dawn water potential | Physiological | Scholander bomb (PMS Instrument, Albany, OR, USA). | 01/11/2020 - 15/04/2021 | Monthly |

**Supplementary Table S2**. Description of all the studied variables with the method, period and frequency of data collection. All data are quantitative.

|  | Photosynthesis | | | Conductance | | | Sapflow | | | Water potential | | |
| --- | --- | --- | --- | --- | --- | --- | --- | --- | --- | --- | --- | --- |
|  | DF | F-value | p-value | DF | F-value | p-value | DF | F-value | p-value | DF | F-value | p-value |
| treatment | 1 | 1.54 | 0.2245 | 1 | 3.01 | 0.093 | 1 | 0.0003 | 0.9861 | 1 | 1.01 | 0.3449 |
| genotype | 4 | 5.56 | **0.0009** | 4 | 2.46 | 0.0652 | 4 | 1.605 | 0.191 | 4 | 0.91 | 0.4141 |
| time | 2 | 221.44 | **0.0001** | 2 | 521.94 | **0.0001** | 2 | 32.29 | **0.0001** | NA | NA | NA |
| season | 4 | 95.6 | **0.0001** | 4 | 19.63 | **0.0001** | 4 | 33.21 | **0.0001** | 4 | 17.79 | **0.0001** |
| treatment:genotype | 4 | 1.21 | 0.3141 | 4 | 0.42 | 0.7904 | 4 | 0.95 | 0.4413 | 4 | 0.83 | 0.5144 |
| treatment:time | 2 | 2.68 | **0.0301** | 2 | 0.53 | 0.5864 | 2 | 0.16 | 0.8517 | NA | NA | NA |
| genotype:time | 8 | 6.68 | **0.0001** | 8 | 4.19 | **0.0001** | 8 | 2.41 | **0.0158** | NA | NA | NA |
| treatment:season | 4 | 3.09 | **0.0018** | 4 | 3.52 | **0.0072** | 4 | 0.65 | 0.6236 | 4 | 4.56 | **0.0014** |
| genotype:season | 16 | 3.77 | **0.0001** | 16 | 2.53 | **0.0008** | 16 | 2.84 | **0.0005** | 16 | 0.7 | 0.7881 |
| time:season | 8 | 19.26 | **0.0001** | 8 | 15.69 | **0.0001** | 8 | 5.08 | **0.0001** | NA | NA | NA |

**Supplementary Table S3.** ANOVA results of the physiological variables (photosynthesis, conductance, sapflow, water potential) for the effects treatment, genotype, time, season, and their interactions. Bold p-values are under 0.05 and therefore significant.
